# Streptozotocin-induced type 1 and 2 diabetes in rodents: a model for studying diabetic cardiac autonomic neuropathy

**DOI:** 10.4314/ahs.v21i2.30

**Published:** 2021-06

**Authors:** Olawale Mathias Akinlade, Bamidele Victor Owoyele, Ayodele Olufemi Soladoye

**Affiliations:** 1 Neuroscience and Inflammation Unit, Physiology Department, College of Health Sciences, University of Ilorin, Kwara State, Nigeria; 2 Cardiology unit, Internal Medicine Department, LAUTECH Teaching Hospital, Ogbomoso, Oyo State, Nigeria; 3 Physiology Department, Bowen University, Iwo, Osun State, Nigeria

**Keywords:** Diabetic cardiac autonomic neuropathy, Diabetes mellitus, Heart rate variability, Streptozotocin

## Abstract

**Background:**

Several animal models are continually being developed to study diabetic complication. Several conflicting regimen for diabetes induction exist in the literature with varying dose strength and regimen for different study interest in diabetes. This study aims to show the effect of high dose streptozotocin (STZ) on the one hand compared with multiple low doses after high fat diet induction on diabetic cardiac autonomic neuropathy (DCAN).

**Methodology:**

Eighty-four Wistar rats were used to demonstrate DCAN induction using 2 approaches one for T1DM (STZ 50mg/kg) and the other for T2DM (HFD for 8 weeks with STZ 25mg/Kg daily for five days). DCAN features were assessed using invasive biomarkers, histology patterns and cardiac nerve densities.

**Results:**

Diabetes induction rate was 76% and 89% in T1DM and T2DM model respectively. T1DM group had significant weight loss, reduced c-peptide, and insulin level post induction. The T2DM additionally showed significantly higher total cholesterol and Homeostatic model assessment (HOMA) compared with control. Serum levels of catecholamine, choactase, nerve growth factor and cardiac nerve density confirms development of DCAN.

**Conclusion:**

High single dose of STZ and HFD with multiple low doses of STZ may be recommended for DCAN study in T1DM and T2DM rat model respectively.

## Background

Diabetes is a chronic metabolic disorder with increasing prevalence and cardiovascular morbidity and mortality^1^–^3^. Several animal models are continually being developed to study the pathogenic mechanism and therapeutic interventions since its complications continue to plague the human race. Streptozotocin (STZ) is a chemical that has been used commonly for the induction of diabetes in experimental models of mice and rodents. It was first reported to have diabetogenic effect in 1963 ever since several studies have used it in different combinations for the induction of diabetes^4^–^7^.

## Mechanisms of STZ action

**Figure 1:**
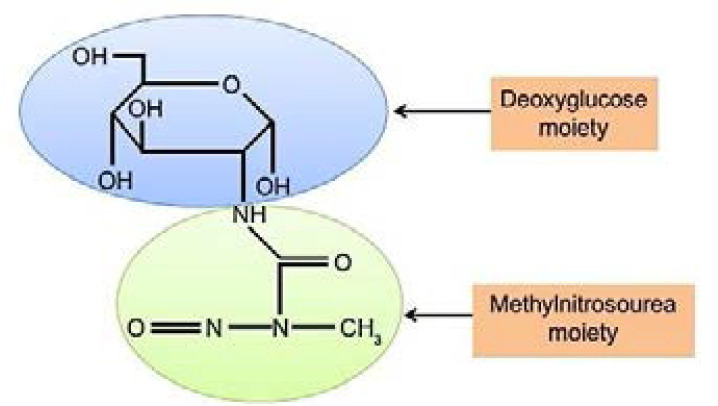
Chemical structure of streptozotocin (Adapted from Wu et al^10^).

STZ is a broad spectrum antibiotic produced by bacterium Streptomyces achromogens^8^. It contains a glucose molecule linked to a highly reactive methyl-nitrosourea moiety that is thought to exert STZ's cytotoxic effects, while the glucose moiety directs the chemical to the pancreatic β cells^8^ (Figure 1). STZ has a short halflife, due to rapid metabolism in the liver and elimination by renal excretion^9^. Once STZ is eliminated out of the body, further functional impairment of the liver or the kidney may be attributed to the effects of diabetic hyperglycemia. This is the basis for studying the mechanisms of STZ diabetic complications in these organs as well as other organs such as te brain, the heart, and the muscles^9^.

## STZ and diabetes induction

There are several conflicting dose regimen for either type 1 and type 2 diabetes induction in the literature. While most researchers hold the fact that type 1 diabetes can be induced in rodents by a single high dose STZ injection, few others make use of multiple doses. Type 2 diabetes, on the other hand, can be induced by several documented approaches, this include STZ injection alongside the administration of nicotinamide^11^, high fat diet (HFD) feeding followed by a low-dose multiple STZ injections, and STZ injection during the neonatal period^4^,^5^,^7^,^12^–^16^.

T2DM models have been previously induced using a combination of HFD and STZ injection. However, there are differences in the food type, duration of the HFD, and STZ injection doses. For instance, Zhang et al., fed rats with chow for 4 weeks after which asingle injection of 45 mg/kg STZ or two STZ (30 mg/kg at weekly intervals for 2 weeks) injections(17). Other reports described feeding male Sprague-Dawley rats normal chow or an HFD for 2 weeks and then injecting 50 mg/kg STZ, while in another study, a combination of an HFD for 2 weeks and then 35 mg/kg STZ as a single injection was used to develop a T2D rat model (5,17,18). In this study, Wistar rats were fed a high-fat and high-carbohydrate diet for 8 weeks followed by the application of low doses of STZ for the development of a diet-induced T2DM animal model.

This study, therefore, aims to show the effect of high dose STZ on the one hand compared with multiple low doses after high fat diet induction on cardiac autonomic neuropathy.

## Materials / Method

### Animals

The study was done in accordance with the guiding principles of the University of Ilorin Ethical Review Committee (UERC). The research was approved by the same Ethical Review Committee (UERC) with approval no: UERC/ASN/2019/1912. Male Wistar rats were used for the experiment. All animals were acclimatized to their environment for 2 weeks before the commencement of the experiments. They were fed ad libitum and housed in pairs in wooden cages.

### Experimental groups/ Model development

**Phase 1:**

Eighty four male Wistar rats were divided into two broad groups, the first was the type 1 model, and the second the type 2 model.

**Type 1 model:** Forty-two male Wistar rats were grouped into two; sham control group (n = 10) and diabetic group (n =32). Type 1 DM was induced with high dose STZ at 50mg/Kg single dose under ketamine anaesthesia. Rats with plasma glucose >16mmol/L on the third day after STZ-injection were randomized into different groups. Whole blood was taken from the proximal ventral tail vein for glucose measurement using a glucometer (Accu-Chek II Boehringer Mannheim Canada, Dorval, Quebec). Fasting plasma glucose levels, water intake, and body weights were recorded at weekly intervals while other parameters were determined at the beginning and end of the experiment.

**Type 2 model:** Type 2 DM was induced with 8 weeks feeding with high fat diet (composition as shown in table 1) thereafter, low dose STZ at 25mg/Kg dose under ketamine anaethesia over 5 days. Ten days after STZ-injection, rats with plasma glucose >16mmol/L were randomized into different groups. Whole blood was taken from the proximal ventral tail vein for glucose measurement using a glucometer (Accu-Chek II Boehringer Mannheim Canada, Dorval, Quebec). Fasting morning plasma glucose levels, water consumption, and body weights were determined at weekly intervals while other parameters were determined at the beginning and end of the experiment.

**Table 1:** Compositions of the control and high-fat diets

Dietary components	Control diet	High-fat diet
**Energy (Kcal/g)**	3.00	6.4
**Calorie percentage**		
**Carbohydrate**	60	30
**Fat**	15	65
**Protein**	25	5
**Weight percentage**		
**Carbohydrate**	15	40
**Fat**	25	45
**Protein**	60	15
**Materials**	standard chow diet	maize, wheat offal, groundnut cake, soya meal, palm kernel cake/oil, bone meal, methionne, lysine, salt, finisher premix, cluppled

### Invasive assay

The rats were humanely euthanized using cervical dislocation after been anaesthetized, blood samples were collected via cardiac puncture after the opening of the upper abdominal region. The blood samples were centrifuged at 1500 g x 15 minutes and the plasma samples micro-pipetted into plain bottles and immediately stored at a temperature of -4°C.

For the invasive assay, protein expression markers, which have been correlated with CAN and diabetes complications in previous studies and also recommended by the CAN subcommittee of the Toronto autonomic neuropathy working group, were assayed^19^–^23^. These were then correlated with the time varying analysis.

### Histological studies

The sections of the rat organs were taken including the pancreas, kidney, liver, and heart. Paraffin sections from organs (3µm thickness) were cut and evaluated using standard staining protocol for H&E and histo-chemistry^24^. Cardiac nerve density was estimated using image J morphometric analysis after standard staining techniques.

### Statistical Analysis

Data was analyzed using Statistical Package for Social Sciences (SPSS) software (Version 23.0; SPSS Inc, IL., USA) for windows. Results were expressed as the mean ± SEM. Group means for two independent samples was compared using Student's t-test and p values less than 0.05 were taken as statistically significant.

## Results

### Diabetes induction and laboratory parameters

**Figure 2:**
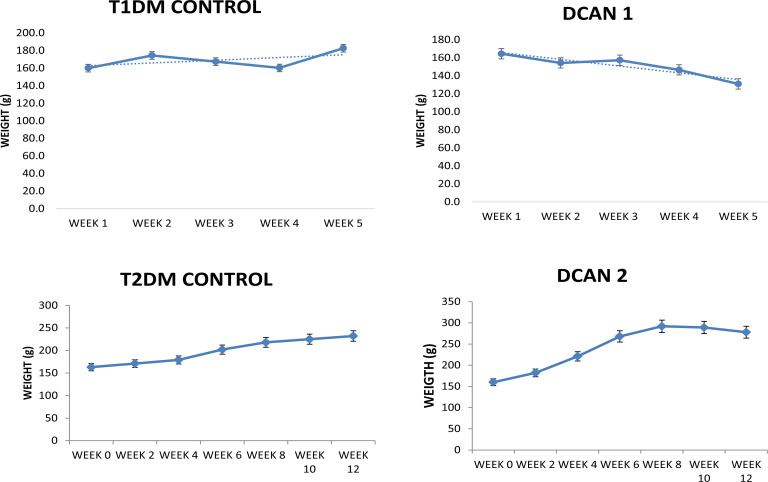
Weight changes in the control and diabetic rat models. T2DM: Animals progressively increased in weight while been fed high fat diet for first eight weeks, later lost weight after STZ administration and DM induction. T1DM: There was progressive weight loss following STZ induction in the DCAN group.*p < 0.05 compared with normal control

This study shows that STZ-induced diabetic rats developed significantly higher glucose level compared with the controls (Figure 2, Table 1).

Type 1 DM model: In the type 1 arm of the study, there was progressive weight loss after administration of 50mg/Kg of STZ at a single dose compared to the control that had sustained weight gain. Seventy-six percent (76%) of the animals in this group developed DM (blood glucose>16mmol/L). The lipid profile, c-peptide, insulin, HOMA, and advanced glycated end products (AGEs) are shown in table 2. Our study showed that for the type 1 model there was no significant derangement in the lipid parameters and HOMA (a measure of insulin resistance).

**Table 2:** Comparison of the laboratory parameters of DCAN and control group

Parameters	Type 1 control	Type 1 post-DM induction	p value	Type 2 control	Type 2 Post-DM induction	p value
**FBS (mmol/l)**	**5.18±0.46**	**17.46±7.84**	<0.0001*	**5.31±0.57**	**18.39±10.47**	<0.0001*
TC (mg/dl)	61.85±8.02	62.89±6.80	0.317	**51.61±8.02**	**69.28±6.40**	0.001*
**TG (mg/dl)**	**66.07±5.64**	**81.64±4.2**	0.047*	**66.07±5.64**	**94.02±7.6**	0.012*
**AGEs (ng/ml)**	**23.04±5.17**	**63.54±2.09**	<0.0001*	**66.04±6.37**	**143.61±9.09**	<0.0001*
C-peptide (ng/ml)	1.13±0.60	0.63±0.53	0.160	**1.20±0.67**	**0.73±0.43**	0.001*
**Insulin (µIU/ml)**	**2.24±1.01**	**0.56±0.47**	0.003*	**2.89±1.01**	**1.14±0.67**	<0.0001*
HOMA-IR	0.12±0.05	0.15±0.04	0.116	**0.13±0.08**	**0.28±0.09**	0.016*
**NGF (pg/ml)#**	**270.16**	**120.56**	0.03*	**234.16**	**150.56**	<0.04*
	**(111.18–318.34)**	**(83.27–160.58)**		**(91.18–311.34)**	**(89.27–169.58)**	
**NA (pg/ml)**	**119.62±15.5**	**589.74±99.48**	0.010*	**113.4±57.65**	**508.6±130.6**	0.010*
**Choactase** **(ng/ml)**	**1.24±0.04**	**0.81±0.03**	0.02*	**1.22±0.04**	**0.93±0.03**	0.03*

Type 2 DM model: This arm of the study showed progressive initial weight gain following high fat diet in the DCAN group compared with the control group, the weight, however, was noticed to decrease after STZ induction. Eighty-nine percent (89%) of the animals in this group developed DM (blood glucose>16mmol/L). The plasma levels of insulin and c-peptide were significantly lower compared with the control (p<0.0001 and 0.001 respectively). Total cholesterol was also seen to be significantly higher in the DCAN group compared with the control (p=0.001). In addition, HOMA, which is a method for assessing β-cell function and insulin resistance (IR) from basal (fasting) glucose and insulin or C-peptide concentrations were also significantly higher in the DCAN group compared with control (Table 2).

There was a significant increase in nor-adrenaline activity (p=0.010), with reductions in nerve growth factor (p<0.0001) and choline acetyl-transferase (CHOACTASE) (p=0.030) compared with the control group (Table 2). Above findings further support the development of DCAN in both T1DM and T2DM model.

### Oral and Intra-peritoneal glucose tolerance test

**Figure 3:**
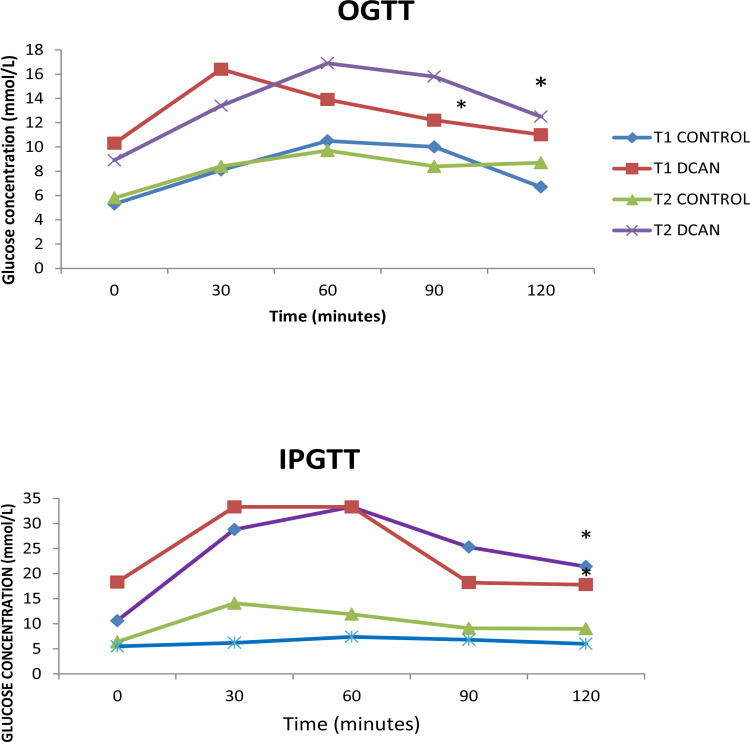
Showing oral and intra peritoneal glucose tolerance test. Values are expressed as Mean ± S.E.M(n=5). * p< 0.05 compared with control. IPGTT= Intra-peritoneal glucose tolerance test OGTT= Oral glucose tolerance test

At the end of the study, both oral and intraperitoneal glucose tolerance tests were done to observe and compare the trend using the different routes of glucose administration. Oral glucose tolerance test result shows an immediate increase in the blood glucose level in both DCAN group and controls. However, there was a significant impairment of glucose handling at 30 min post glucose ingestion and further thereafter. Blood sugar level decreased in the next 60, 90, and 120 min time intervals in the control group, while it increased in the DM group (figure 3). Besides, DM group indicated a glucose tolerance defect that could be a sign of disability in absorbance and response to glucose (which further corroborates DM induction). The intraperitoneal administration of glucose also shows significant impairment in glucose homeostasis howbeit, more impairment than when administered through the oral route.

### Histology of Pancreas

**Figure 4:**
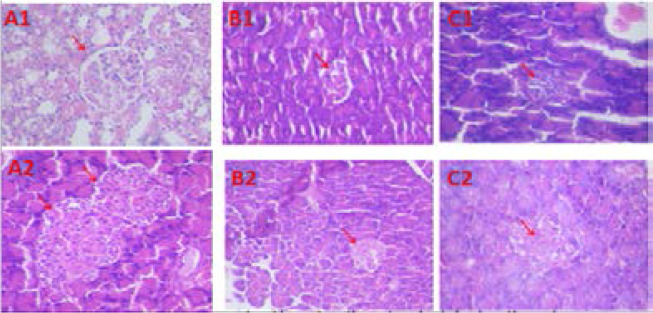
Photomicrographs of hematoxylin and eosin stained sections of the rat pancreas. A1&2= Control groups with normal glandular cells, B1&2=Type 2 DM group, C1&2=Type 1 DM group with shrinked islets cells, degeneration of B cell nuclei with edema and hemorrhage. Red arrow= Pancreatic islet cells.

Histology sections of the pancreas show normal pancreatic islet cells with deeply-staining nuclei, normal sized blood vessels, and pancreatic ducts in the control group. The exocrine pancreas also showed normal glandular cells with deeply staining nuclei. The type 1 DM model shows significant shrinkage of the islet of Langerhans cells with degeneration of the beta cell nuclei. This is in contrast to the type 2 model which shows oedematous pancreatic tissue with a widening of the inter-glandular spaces and engorged blood vessels with less shrinkage of the islets of Langerhans (Figure 4).

## Discussion

The present study shows induction of diabetes in rat model using STZ alone (T1DM) and in combination with high fat diet (T2DM). High-fat feeding will lead to obesity, hyperinsulinemia, and altered glucose homeostasis due to insufficient compensation by the beta cells of the pancreatic islets^7^. The T2DM model in our study typifies human T2D progression with associated dyslipidaemia, insulin resistance, prediabetes, and diabetes. Diabetes is a complicated, heterogenic, and polygenic disease, while T1DM is associated with destruction in the pancreatic acini cells commonly via auto-immune mechanism. T2DM is associated with the development of insulin resistance by target organs and decreased insulin production from pancreatic beta cells^11^,^15^,^18^,^25^. igh single dose of STZ as seen in this study was associated with sudden and significant destruction of the pancreatic cells as show in the histology slides in figure 3. Accordingly, progressive multiple doses of STZ after HFD causes less destruction of the pancreas, this animal model portraits the same characteristics and mimic the pathogenesis and clinical features of human T2D. Some researchers earlier showed that an HFD for 2–7 weeks would induce stable insulin resistance and the HFD in combination with low doses of STZ induces diabetes in rats^7^,^26^. Here, in our study, we observed a significant increase in the blood glucose and total cholesterol, although some other study showed significantly elevated triglyceride levels^14^,^15^,^27^. This may be in part due to the differences in the composition of the high fat diets used.

Since there were different doses and number of STZ injections reported for the development of T2DM rat model, we tried to using a protocol which will be a similar model to the human disease. While Reed et al. used 50 mg/kg STZ for inducing T2D^14^, Srinivasan et al. however, used 35 mg/kg interperitoneal injection^18^. On the other side, the period of HFD usage was also variable, some protocol used the HFD for 2–4 weeks before STZ injection, Zhang et al. used HFD for 2 months^17^. Based on our results, 8 weeks HFD decreases the insulin secretion by pancreatic cells as well as the induction of insulin resistance by target tissues.

Our study also showed differences in the response of plasma glucose levels between IPGTT and IGGTT in STZ induced animal model. The peak blood glucose level in both T1DM and T2DM model following bolus glucose challenge was higher in IPGTT than IGGTT. This was similar to findings by Watada et al and Andrikopoulos et al possibly implying^28^,^29^ that a major amount of glucose administered through the gastric route is taken up by the splanchnic bed during its first pass through the organs. Moreover, earlier researchers have reported that glucose ingestion during oral glucose tolerance tests (OGTT) stimulated more GLP-1 production than during intravenous glucose tolerance test (IVGTT) in patients with type 2 diabetes, thus intragastrically administered glucose may be more efficient in stimulating insulin release from pancreatic beta cells for lowering blood sugar level^30^.

Choline acetyl-transferase (a marker of parasympathetic nerve activity) was significantly lower in the DCAN group compared with the control. This was similar to findings by Yang et al which showed that homogenized cardiac tissue had lower choactase levels and tyrosine hydroxylase^31^. He further showed that the reduced choactase correlated with lower western blot choactase antibodies and autonomic nerve rarefaction. This further supports the development of cardiac autonomic neuropathy in these animal models.

Our study showed a similar trend with regards to the STZ effect on pancreatic tissues and other earlier findings^32^,^33^. For instance, Mythili et al showed that 30-mg/kg body weight STZ produces mild changes in pancreatic tissues while 50 mg/kg proves to be fatal^32^. The doses used were similar to our type 2DM (25mg/Kg) and T1DM (50mg/Kg) models respectively. Findings in this study also corroborates the nearly complete destruction of the beta cells as found in type 1 DM patients, while T2DM patients still have preserved residual beta cells till very late in the disease spectrum.

In resource constraint research laboratories, the development of transgene T2D animal models still remain technically complicated, time consuming and costly, diet manipulation would be an ideal alternative. Since the protocols presented in the literature for stable diet-induced T2DM animal models are heterogeneous with no consensus existing for a standard procedure, conducting studies in this field to reach a common procedure for T2DM animal model production is of intensive value. Accession to an easy, cost-effective, and accurate animal model would facilitate more studies to unravel pathogenic mechanism of the disease while also developing newer therapeutic interventions in diabetes.

## Conclusion

This study, therefore, shows that the association of HFD with multiple low doses of STZ administered intra-peritoneally and single high dose of STZ in adult animals was effective in inducing T2DM and T1DM in rats respectively. These models are proposed for studying cardiac autonomic neuropathy in rodents in resource constraint settings where transgenic models may be technically difficult to develop.

## Declarations

### Availability of data and materials

The datasets during and/or analysed during the current study available from the corresponding author on reasonable request.
